# Determination of Chemical Constituents and Antioxidant Activities of Leaves and Stems from *Jatropha cinerea* (Ortega) Müll. Arg and *Jatropha cordata* (Ortega) Müll. Arg

**DOI:** 10.3390/plants10020212

**Published:** 2021-01-22

**Authors:** Yeimi Cecilia Vega-Ruiz, Corina Hayano-Kanashiro, Nohemí Gámez-Meza, Luis Angel Medina-Juárez

**Affiliations:** Departamento de Investigaciones Científicas y Tecnológicas de la Universidad de Sonora, Blvd. Luis Donaldo Colosio s/n, Entre Reforma y Sahuaripa, Edificio 7G, Col. Centro, Hermosillo, Sonora C.P. 83000, Mexico; yeimi_cecilia@hotmail.com (Y.C.V.-R.); angela.hayano@unison.mx (C.H.-K.); nohemi.gamez@unison.mx (N.G.-M.)

**Keywords:** *Jatropha*, phytochemicals, antioxidant activity, phenolic compounds

## Abstract

*Jatropha* species have been shown to be an important source of secondary metabolites with different biological effects. *Jatropha cinerea* (Ortega) Müll. Arg and *Jatropha cordata* (Ortega) Müll. Arg are distributed in the Northwestern region of Mexico, are adapted to extreme weather conditions and are widely used (stems, leaves, and sap) in traditional medicine. The aim of the present study was to carry out the phytochemical characterization and the evaluation of the antioxidant activity in methanolic extracts of stems and leaves from *J. cinerea* and *J. cordata*. The compounds present in the extracts of both species were characterized by ESI-IT-MS/MS and quantified by HPLC-DAD. The results showed that the stem extracts of both species are rich in phenolic acids, while the leaf extracts are rich in flavonoids. Some of the main compounds found were gallic acid, gentisic acid, 3,4-Dihydroxybenzoic acid, vitexin, isovitexin, and catechol. Both species showed high concentrations of phenols and total flavonoids and antioxidant activity. *J. cordata* showed the highest antioxidant capacity and the highest concentration of phenolic compounds. Overall, both *Jatropha* species are a natural source of antioxidant compounds with potential biotechnological uses.

## 1. Introduction

The Euphorbiaceae family is considered one of the most diverse, with around 7800 species [1]. Among the genera that belong to this family is *Jatropha* L. This genus is widely distributed in tropical and subtropical regions from Africa, America, and Asia. Mexico is the diversity center and endemism of the *Jatropha* genus. A total of 50 of the 186 known *Jatropha* species are found in Mexico (even in areas with extreme temperature and humidity conditions) and 39 of them are endemic [2,3].

*Jatropha* species have been used for different purposes; one of them is to produce biodiesel, being the seed oil of *Jatropha curcas* L., the main product at an industrial level [4]. In Mexico and other countries, the *Jatropha* species such as *Jatropha curcas* L. (Peru, Ghana), *Jatropha multifida* L. (Cambodia, Nigeria), *Jatropha gossypiifolia* L. (India, Brazil), *Jatropha gaumeri* Greenm (México) and *Jatropha elliptica* M. Arg (Brazil) are used as traditional medicine. When these *Jatropha* species were studied, the results showed that the roots, stems, and leaves of this plant are a source of phenolic compounds and triterpenes. In addition they showed antioxidant, anti-inflammatory and antimicrobial activity [5]. Nowadays, the knowledge generated from *Jatropha curcas* L. studies could contribute to the global development of other *Jatropha* species. Therefore, it is important to perform studies that evaluate the potential of the genetic resources of all Mexican species of this genus.

There are four *Jatropha* species: *Jatropha cinerea* (Ortega) Müll. Arg, *Jatropha cordata* (Ortega) Müll. Arg, *Jatropha cardiophylla* (Torr.) Muell.-Arg, and *Jatropha cuneata* Wiggins and Rollins, widely distributed in the most arid region of Northwestern Mexico. In addition, these four species are considered within the Priority Terrestrial Regions of the National Commission for the Knowledge and Use of Biodiversity (CONABIO) [6,7].

*J. cinerea*, commonly known as “sangrengado”, is widely distributed along the Baja California peninsula, and the coasts of Sonora and Sinaloa, Mexico [8]. A *J. cinerea* shrub is characterized by its resistance to long periods of drought and its development in saline soils and growth in hot, as well as in dry and semi-dry conditions at 1500 m above sea level too; they can measure between 1 to 3 m high, they have thin stems, kidney-shaped leaves, pink flowers with tubular or globular appearance and rounded fruits. The steam bark is brown to whitish in color. It has shallow roots and appears as succulent roots above the ground. The leaves are 2 to 6 cm wide; they are more or less cordate at the base, and its fruit is 1.5 to 2.5 cm wide [6,9].

*J. cordata* is a perennial tree that can grow up to 10 m of height. It is distributed in the arid and semi-arid regions of Mexico and the southern United States. This plant has a taller than wide appearance, soft wood and semi-succulent. It has a well-developed main trunk and small flexible branches. A particular characteristic of *J. cordata* is that the stem of thin sheets emerges similar to paper, this is the reason why this it is called “papelillo”. The leaves are 2.5 to 6 cm wide, with its fruit being 1.5 to 2.0 cm wide [10,11,12].

On the other hand, ethnobotanical studies, it has been reported that *Jatropha* species have been used to treat diseases. The roots of *J. cinerea* are used by the Seri ethnic group in the state of Sonora (Mexico) to cure dysentery and the sap to treat mouth ulcers [13]. The *J. cordata* root is used by ethnic groups in the state of Sonora to combat toothache and the stem and leaves are used to cure gums [14].

Phytochemical studies of the genus *Jatropha* have increased in recent years due to the high potential of these species as natural sources of bioactive compounds. The compounds isolated from *Jatropha* species are phenolic acids, lignans, flavonoids, coumarins, alkaloids, and terpenes, which have shown antioxidant, cytotoxic, antimicrobial, antifungal, and anti-inflammatory activities [15]. However, these secondary metabolites and their biological effect have been studied just in few *Jatropha* species [16].

For this reason, it is important to carry out the first phytochemical study of the stem and leaves of *J. cinerea* and *J. cordata*. The information obtained in this study will be useful to highlight the importance of these endemic species in the arid region of Northwestern Mexico as a potential source of bioactive compounds.

## 2. Results and Discussion

### 2.1. Qualitative Analysis of Phytochemical Compounds

The compounds present in the studied fractions were flavonoids, phenols, coumarins, sterols, and tannins (Table 1). The alcoholic fractions of *J. cinerea* presented flavonoids, phenols, coumarins, and tannins; while the alcoholic fractions of *J. cordata* only presented flavonoids, phenols, and tannins. There are reports of other *Jatropha* species showing a high content of phenolic compounds [17,18]. On the other hand, coumarins in ethyl acetate and alcoholic fractions of *J. cinerea* were found. Additionally, the presence of sterols was detected in the hexane fractions.

Results of alcoholic fractions from the aerial part of *J. cinerea* and *J. cordata* are important because recent publications have reported that alcoholic extracts of these species are used for medicinal purposes [19]. Furthermore, for other species belonging to the genus *Jatropha*, qualitative phytochemical profiles like those presented in the methanolic extracts of the aerial part of *J. cinerea* and *J. cordata* have been reported. Phenols, flavonoids, and tannins were reported in the alcoholic extracts from *Jatropha mollissima* (Pohl) Baill leaves [20]. On the other hand, *Jatropha curcas* L. alcoholic extracts were rich in phenolic compounds [17,18].

### 2.2. Phenol and Flavonoid Content

*J. cinerea* and *J. cordata* leaf extracts showed a higher concentration of phenols and flavonoids than those of stems extracts. Comparing the two *Jatropha* species, *J. cordata* extracts from leaves and stems showed higher phenols and flavonoids contents (Table 2).

The content of phenolic compounds in leaves and stems from *Jatropha curcas* L. were lower than the values reported in this study. *Jatropha curcas* L. plant collected from Malaysia showed a total phenol content in leaves of 1.33 ± 0.013 mg GAE/g and 0.11 ± 0.013 mg GAE/g in stems [21]. Namulli et al. [22] also found that *Jatropha curcas* L. leaves and stems (collected in Malaysia) showed lower total phenols (9.29 mg GAE/g, 3.09 mg GAE/g, respectively) and flavonoids contents (0.91 mg Rutin E/g, 0.38 mg Rutin E/g, respectively), than the ones reported in the present work. On the other hand, El-Baz et al. [23] reported that leaves methanolic extract of *Jatropha curcas* L. from Egypt showed a lower content of total phenols (51.25 mg GAE/g) than the values obtained in the present study; being *J. cinerea* and *J. cordata* native from the most arid region of Northwestern Mexico. Recently, the total phenol content in methanolic extracts from *Jatropha mollisima* (Pohl) Baill leaves, an endemic species from Brazilian semiarid region, was reported with values around 245.12 mg GAE/g [20]. Maybe, different environmental conditions could influence the total phenolic content in *Jatropha* species, as observed in wild *Jatropha mollisima* (Pohl) Baill and cultivated *Jatropha curcas* L. There are reports showing the influence of weather and others external factors on the synthesis of these secondary metabolites [24,25]. Further, the phenolic compounds profile could vary among same species even if different factors are involved, such as genotype, phenological state of the plant and time of cultivation [26,27]. All of these factors could explain the variations found in the phenolic compounds and their concentration in leaves and stems from different *Jatropha* species.

### 2.3. Antioxidant Activity

Extracts of leaves and stems from *J. cinerea* and *J. cordata* showed antioxidant activity. However, the antioxidant activities (IC50 values) of the *J. cordata* leaf and stem extracts, were higher than those of the *J. cinerea* extracts. Phenolic compounds have the capacity to donate one or more of their additional electrons to free radicals; therefore they are excellent free radical scavengers. For example, flavonoids donate electron from their dihydroxy substituents at the 3′ and 4′ position of the B ring of their molecule, neutralizing free radicals or chelating metal ion [28].

In the present work, the antioxidant activity in both wild *Jatropha* species was evaluated by ABTS and DPPH, to analyze the simple electron transfer (SET) mechanism of phenolic compounds. As observed, the IC50 values of the *Jatropha* leaf and steam extracts were different in both radicals used. (Table 2). Physical and chemical properties of the alcoholic extracts, the solvent system and the pH used can lead to different kinetics and side reactions [29].

### 2.4. Identification of Phenolic Compounds by the Extract ESI-IT-MS-MS

**Figure 1:**
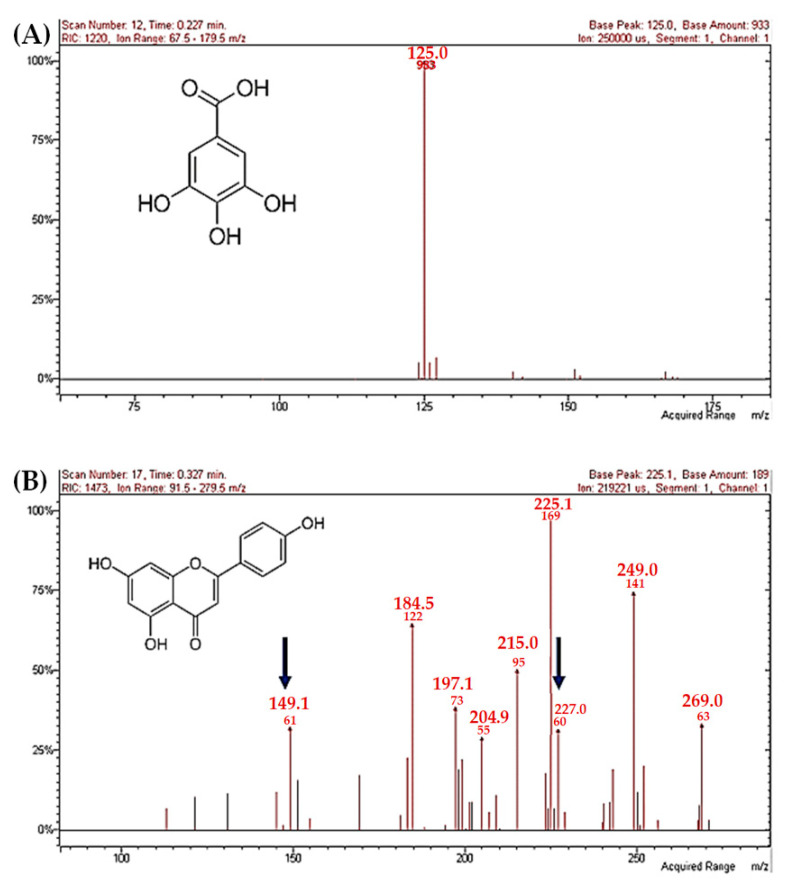
Fragmented ion of two phenolic compounds found in leaves and stems from *J. cinerea* and *J. cordata* by mass spectrophotometer with electrospray in negative ionization mode and ion trap (ESI-IT-MS/MS). (**A**) Gallic acid. (**B**) Apigenin.

Mass spectroscopy methodology was used to determine the different phenolic compounds, present in methanolic extracts from leaves and stems of *J. cinerea* and *J. cordata*. This methodology allows us to identify these compounds through the fragmentation of the molecules in the sample [30]. Therefore, the phenolic compounds were confirmed by comparing them with pure standards and using data from other references (Figure 1).

The phenolic compounds present in the leaves and stems from *J. cinerea* and *J. cordata* are showed in Table 3. The phytochemical compounds profile identified in the alcoholic extracts of leaves and stems from *J. cinerea* and *J. cordata* is similar to that already reported for *Jatropha gossypiifolia*, being *p*-coumaric, ferulic, caffeic, gallic, vanillic, and gentisic acids, catequin, vitexin, isovitexin, luteolin and apigenin the main compounds [31,32]. Respect to flavonoids, *J. cinerea* and *J. cordata* presented quercetin and rhoifolin, which are the same compounds reported in *Jatropha curcas* L. [15].

### 2.5. HPLC-DAD Analyses of Methanolic Extracts

Several compounds belonging to the main phenolic subclasses identified by means of mass spectrometry analysis in the methanolic extracts of leaves and stems from both *Jatropha* species were quantified by HPLC-DAD and their concentration was expressed as mean ± SD (*n* = 9) and summarized in Table 4.

The quantified compounds present in leaves extracts from *J. cinerea* were the phenolic acids such as acids 4-hydroxybenzoic, caffeic, gallic, gentisic, *p*-coumaric, and the flavonoid apigenin, while in the stem extracts were 4-hydroxybenzoic, caffeic, gallic, gentisic, *p*-coumaric, syringic and vanillic acids, and the flavonoids found were apigenin, epicatechin, and quercetin. The compounds quantified in *J. cordata* leaves extracts were 4-hydroxybenzoic, caffeic, gallic, gentisic and *p*-coumaric acids, and flavonoids apigenin and quercetin, while in stems were 4-hydroxybenzoic, caffeic, gallic, gentisic, sinapic and vanillic acids, and the flavonoids apigenin, epicatechin, and quercetin. Data showed that the contents of each phenolic compound from the leaves and stems of *J. cinerea* and *J. cordata* were significantly different between species and plant organs.

According to the results, the phenolic compounds profile in both *Jatropha* species indicates that they are not uniformly distributed throughout the plant. It is suggested that some compounds are more concentrated in the roots and seeds and others in the green tissues of the aerial part (stems and leaves). This can be possible because each organ has a specialization that must fulfill according to its physiological function, which generated differences in the phenolic compounds profile [40]. For example, it was reported that leaf has the ability to synthesize phenolic metabolites as a rapid response to external stimuli such as microbial attack and light [41]. Furthermore, it is well known that the synthesis of secondary metabolites depends on the season and plant growth stage [42].

Flavonoids represented the highest concentration of phenolic compounds in stem methanolic extracts from *J. cinerea* and *J. cordata*, while leaf extracts showed the highest concentration to phenolic acids. Comparing extracts of both species, *J. cordata* was the species with the highest concentration of phenolic compounds, being gentisic acid, the phenolic acid with the highest concentration (864.62 µg/g), and quercetin as the most abundant flavonoid presented in the extracts, with a concentration of 573.98 µg/g. Extracts of stems and leaves from *J. cinerea,* also presented gentisic acid as the most abundant phenolic acid with a concentration of 634.55 µg/g; while the most abundant flavonoid was epicatechin (230.05 µg/g). In addition, these results showed a positive correlation with the antioxidant activity of the methanol extracts from leaves and stems of *J. cinerea* and *J. cordata* (Table 2).

Another important phenolic compound is the gallic acid (3,4,5-trihydroxybenzoic acid), which is present in the plant kingdom and is capable of exhibiting antioxidant properties [43]. Gallic acid was present in the methanolic extracts of leaves and stems from *J. cinerea* and *J. cordata* in higher amounts than those reported in methanolic extracts for *Jatropha curcas* L. leaves (220 µg/g) [22].

Gentisic acid (2,5-dihydroxybenzoic acid) is the phenolic acid with the highest concentration in the leaves of *J. cinerea* and *J. cordata* (Table 4). This is a diphenolic compound and a derivative of benzoic acid [44]. Gentisic acid can be found in plants such as grapes (*Vitis vinifera*) and citrus fruits (*Citrus* spp.). This compound has shown to have activity as antioxidant, anticancer, hepatoprotective, antimicrobial, and antiinflammatory. Furthermore, no strong evidence of adverse effects and toxicity of gentisic acid has been reported [45].

In the present study within flavonoids identified, the apigenin was found in greater quantity in the leaves of *J. cinerea* and *J. cordata* (Table 4). Apigenin chemical name is 4′, 5, 7, -trihydroxyflavone, is a nontoxic active biological flavonoid and it has been detected in some fruits such as grapefruit, onions, oranges, tea, chamomile, and in some seasonings [46]. It was recently reported that apigenin has the antioxidant and antiinflammatory properties and can be used as a natural product in reducing some of the symptoms of polycystic ovary syndrome [47].

## 3. Materials and Methods

### 3.1. Chemicals and Reagents

All reagents, solvents and chemicals standards were acquired from Sigma Aldrich Co. (St Louis, MO, USA). The solvents used for high-performance liquid chromatography (HPLC) analyses were methanol and formic acid from JT Baker (Xalostoc, Mexico State, Mexico) and Milli-Q water (EMD Millipore Corporation, Billerica, MA, USA).

### 3.2. Plant Material

**Figure 2:**
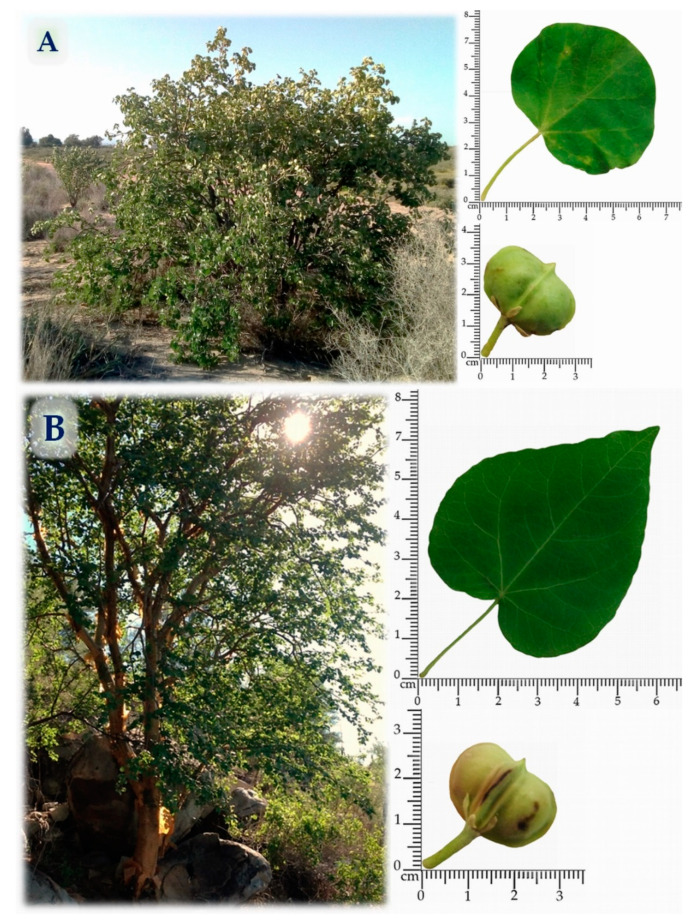
*Jatropha* species, leaves and fruits. (**A**) *Jatropha cinerea* (Ortega) Müll. Arg, (**B**) *Jatropha cordata* (Ortega) Müll. Arg.

The stems and leaves from *J. cinerea* and *J. cordata* were collected from the central area of Sonora state in arid region of northwestern Mexico (Figure 2). *J. cinerea* was collected from the coastal area of Hermosillo city during August 2018 (N 28°49.831′, W 111°56.672′), while *J. cordata* was collected from the periphery area from Hermosillo city, during September 2018 (N 29°10.994′, W 110°55.846′). For each *Jatropha* species, the sample were randomly collected from at least 18 individuals, taking around 150–200 g of leaves and stems. The material was kept in paper bags and was protected from light and moisture until it was dried.

The taxonomic identification of both *Jatropha* species was confirmed by experts in the Herbarium of the University of Sonora (Sonora, Mexico).

### 3.3. Preparation of Sample

The leaves and stems of both species were manually separated. The samples were dried at room temperature (23 ± 2 °C) for 3 months and protected from the light and moisture. The material was grinded with a blender (Oster model 4122, Mexico City, Mexico) until powder and it was sieved at particle size of 500 µm.

### 3.4. Extraction of Phenolic Compounds

The phenolic compounds extraction was performed according to Molina-Quijada et al. [48]. The sample (1 g) was mixed with 10 mL of methanol:water (70:30, *v*/*v*), shaken vigorously in a vortex, and sonicated (Sonic 1510 R-DTH, Branson Ultrasonics Corporation, Danbury, CT, USA) for 30 min. Then, the mixture was centrifuged (3000× *g*) for 15 min at 4 °C (Centrifuge IEC CL3 IR, Thermo Electron Industries SAS, Chaâteau-Gontier, Mayenne, France). The methanolic extraction was repeated twice at room temperature (23 ± 2 °C), protecting samples from light. A total extraction volume of 20 mL was obtained for each gram of sample. The supernatants were recollected, filtered (Whatman membrane filters nylon, pore size 0.2 µm) and stored at −20 °C until further analysis.

### 3.5. Qualitative Analyses of Phytochemical Compounds

The phytochemical compounds present in methanolic extracts of aerial part (stems and leaves) from the two species of *Jatropha* were screened by qualitative assays of the secondary metabolites. The solvents of the extracts of each species were evaporated under reduced pressure at 40 °C, followed by adding consecutively the different solvents from minor to major polarity like hexane, ethyl acetate, ethanol, and methanol (50 mL of each solvent). Qualitative phytochemical tests for alkaloids, glycosides, coumarins, tannins, sterols, quinones, saponins, phenols, and flavonoids were carried out according to Janaki et al. [49]. The color intensity was used as a response of mentioned tests. The results were interpreted such as presence (+) and absence (−) of the phytochemical groups in the fractions.

### 3.6. Determination of Total Phenolic Compounds

The total phenolic content in the extracts from leaves and stems from both *Jatropha* species was carried out according to Ashish et al. [50]. In 96-well plates, 30 µL of extract were placed and 150 µL of diluted Folin–Ciocalteu reagent was added to each well and these were incubated at room temperature for 3 min. Then 120 μL of sodium carbonate were added and they were incubated for 30 min at room temperature protected from light. Finally, the absorbances were measured at 765 nm (Varioskan LUX Multimode Microplate Reader, Thermo Scientific, Vantaa, Finland). The total phenolic compounds content was determined as gallic acid equivalents per g of sample (mg GAE/g), using the linear equation based on the calibration curve.

### 3.7. Determination of Total Flavonoids

The total flavonoids content in the extracts was determined by a colorimetric assay [51]. Then 2 mL of extract were mixed with 300 µL of NaNO_2_ (5%). After 6 min, then 300 µL of AlCl_3_ (10%) were added and after 1 min, 1 mL of NaOH (4%) was added. The final volume was brought up to 6 mL with deionized water. From this mixture, 300 µL of each tube was added to the 96-well plate, reading the absorbance at 510 nm (Varioskan LUX Multimode Microplate Reader, Thermo Scientific, Vantaa, Finland). A quercetin calibration curve was performed to quantify the total flavonoids contents. Results were expressed as quercetin equivalents per gram of sample (mg QE/g).

### 3.8. Antioxidant Activity

The antioxidant activity of the methanolic extracts of the two *Jatropha* species was evaluated by two techniques based on the system of transfer individual electrons (SET), which implies a unique redox reaction, being the oxidant the indicator for end point measurement [29].

#### 3.8.1. DPPH Method Antioxidant Capacity

The DPPH radical (working solution) was prepared mixing 2.5 mg of DPPH reagent (2,2-diphenyl-1-picrylhydrazyl) with 80 mL of methanol. The absorbance of the working solution was adjusted to 0.70 ± 0.02 at 515 nm. The experiment was carried out in 96-well microplates where 20 μL of each sample was added to each well followed by the addition of 180 μL of DPPH working solution. Then, the plate was incubated in the dark during 30 min at room temperature (23 ± 2 °C) [52]. Finally, the absorbance was measured in a microplate reader (Varioskan LUX Multimode Microplate Reader, Thermo Scientific, Vantaa, Finland) at 520 nm and compared against a standard curve of Trolox. Results were expressed as percent of inhibition.

#### 3.8.2. ABTS Method

The 2,2′-azino- bis (3-ethylbenzothiazoline-6-sulfonic acid) ABTS•+ radical cation was prepared by mixing 10 mL of 2.45 mM K_2_S_2_O_8_ with 38.4 mg ABTS reagent. The mix was kept under dark conditions with constant shaking during 12–16 h at room temperature (23 ± 2 °C). The ABTS•+ radical cation was mixed with methanol 70% (*v*/*v* methanol:water) until reach an absorbance of 0.70 ± 0.02 at 734 nm (working solution). In a 96-well microplate, 25 µL of extracts were added to 225 µL of the previously prepared solution. Samples were incubated for 5 min at room temperature (23 ± 2 °C) under darkness. Finally, the absorbance was measured at 734 nm (Varioskan LUX Multimode Microplate Reader (Thermo Scientific, Vantaa, Finland)) [53]. Results were compared against a standard curve of Trolox and expressed as percent of inhibition.

Concentrations of 1.11 to 50 mg/mL of leaves and stems methanolic extracts from *J. cinerea* and *J. cordata* were used. For the assays, the concentration necessary for the inhibition of 50% of ABTS and DPPH radicals was calculated with a lineal regression using the software GraphPad Prism 8. Trolox was used as comparation antioxidant standard.

### 3.9. Identification of Phenolic Compounds by ESI-IT-MS-MS

The identification of compounds present in the leaves and stems extracts of both *Jatropha* species was done injecting the extracts (10 μg/mL *p*/*v*) by direct infusion to a Varian 500-MS mass spectrometer (Walnut Creek, CA, USA) equipped with an electrospray ionization source and an ion trap mass analyzer, which were controlled by MS WorkStation software v.16 (Agilent Technologies, Santa Clara, CA, USA). The mass spectrometer was operated in negative mode, nitrogen as a misting gas and helium as a collision gas for accurate mass measurement at a flow rate of 1 mL/min. Mass spectra were acquired in full scan mode in the range of 100–2000 *m*/*z* using a flow rate of 10 µL/min a voltage −17 KV, and capillary temperature of 350 °C. The manipulation of the extracts was carried out with protection of the light. The identification of several metabolites was performed by second order fragmentation (MS/MS). The confirmation was carried out comparing the fragmented ions with pure standards (compounds 2, 12–14 and 19; Table 3) and using data from bibliography (compounds 1, 13, 15–18, 20–22; Table 3).

### 3.10. HPLC-DAD Analysis of Extracts

**Figure 3:**
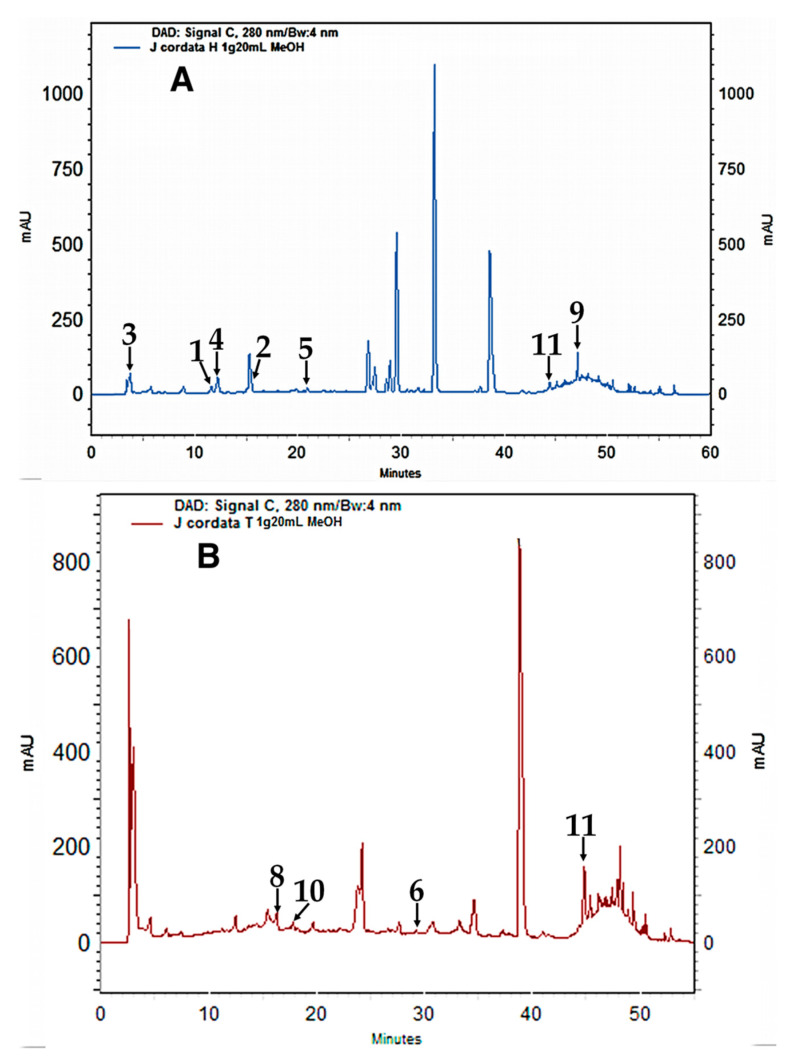
High-Performance Liquid Chromatography chromatograms of *Jatropha cordata* extracts (280 nm). **A**: Leaves extract. **B**. Stems extract. (**1**) 4-Hydroxybenzoic acid, (**2**) Caffeic acid, (**3**) Gallic acid, (**4**) Gentisic acid, (**5**) *p*-coumaric acid, (**6**) Synapic acid, (**8**) Vanillic acid, (**9**) Apigenin, (**10**) Epicatechin, (**11**) Quercetin. For peaks between 30 and 40 min, considering the mass spectrophotometer information, they could correspond to sinapic acid, rutin hydrate and vitexin. However, there was no conclusive evidence for their identification when compared with the retention times of commercial standards.

The quantification of the phenolic compounds presents in the extracts of both *Jatropha* species was performed using an Agilent 1200 liquid chromatography system (Agilent 1260 Infinity and ChemStation Software, Agilent Technologies, Palo Alto, CA, USA) coupled to a diode array detector (HPLC-DAD, Agilent Technologies, Inc., Santa Clara, CA, USA). The separation of the compounds was done on a C-18 HPLC column (5 µm, 25 cm × 4 mm, SupelcosilTM LC-18, SUPELCO). The mobile phase was a gradient of solvents: A (5% formic acid) and B (methanol) to 1 mL/min flow rate [54]. The leaves and stems extracts were filtered (nylon filter, 0.22 µm) and injected in the chromatography system (20 µL of leaves and 50 µL of stems). The chromatograms were recorded at 260, 280, 320, 330, and 370 nm. The identification of compounds was done comparing the peak retention times and UV spectra of the samples against data obtained fron the commercial standards (Figure 3). The compounds were quantified using standard curves of 4-hydroxybenzoic, caffeic, gallic, gentisic, *p*-coumaric, sinapic, syringic and vanillic acids, and flavonoids apigenin, epicatechin, and quercetin were performed with corresponding standards (Table 4). The concentration of each compound present in the extracts of both *Jatropha* species were expressed as µg per g of sample (µg/g) as the mean (*n* = 9) ± standard deviation.

### 3.11. Statistical Analysis

All results were expressed as means ± standard deviation (SD) of three independent experiments. One-way analysis of variance (ANOVA) was carried out using the statistical software R studio, version 1.3.959-1 (StatSoft Poland, Cracow, Poland). Significant differences (*p* < 0.05) were determined by the Tukey’s test.

## 4. Conclusions

The determination of the chemical components carried out in methanolic extracts of leaves and stems from *J. cinerea* and *J. cordata*, showed that both species are an important source of phenolic acids and flavonoids, when compared to studies reported in other *Jatropha* species. Overall results showed further that the contents of each phenolic compound of leaves and stems from *J. cinerea* and *J. cordata* were significantly different between species and plant organs. This can be related to the specialization that each of these organs has must fulfill, its physiological function and the relationship of the phenolics compounds with it. Additionally, the ability of the leaf to synthesize phenolic metabolites is very important because it is related to the response to external stimuli such as microbial attack and light. Furthermore, leaves of *J. cordata* showed the highest antioxidant activity compared with those obtained from *J. cinerea*. On the other hand, it is shown that the stems and leaves of *J. cinerea* and *J. cordata* may have potential as a source of bioactive compounds. Overall, this study shows the potential application of these two endemic species from the arid regions of Northwestern Mexico adapted to extreme temperature and salinity conditions. To verify the bioactive potential of the phytochemicals identified in both *Jatropha* species studied, the evaluation of the cytotoxicity, antimicrobial and anti-inflammatory activity should be carried out in the future.

## Figures and Tables

**Table 1:** Qualitative analysis of phytochemical compounds from aerial part (leaves and stems) of *J. cinerea* and *J. cordata.*

Phytochemicals	*J. cinerea*				*J. cordata*			
	H	EA	E	M	H	EA	E	M
Flavonoids	−	−	+	+	−	−	+	+
Phenols	−	−	+	+	−	−	+	+
Coumarins	−	+	+	+	−	−	−	−
Saponins	−	−	−	−	−	−	−	−
Sterols	+	−	−	−	+	−	−	−
Tannins	−	−	+	+	−	−	+	+
Alkaloids	−	−	−	−	−	−	−	−
Quinones	−	−	−	−	−	−	−	−
Glycosides	−	−	−	−	−	−	−	−

(+) Present, (−) Absent. Solvents; H: hexane, AE: Ethyl acetate, E: ethanol and M: methanol.

**Table 2:** Phenol and flavonoid content and antioxidant activity of methanolic extracts of leaves and stems from *J. cinerea* and *J. cordata.*

	*J. cinerea*		*J. cordata*	
	Leaves	Stems	Leaves	Stems
Total phenols (mg GAE/g)	82.65 ± 1.65 *	62.02 ± 2.80 *	215.63 ± 3.62 *	153.39 ± 2.91 *
Total flavonoids (mg QE/g)	11.30 ± 0.13 *	3.27 ± 0.11 *	18.55 ± 0.23 *	7.41 ± 0.14 *
ABTS IC50 (mg/mL)	23.03 ± 2.31 *	15.92 ± 0.01 *	7.59 ± 1.10	7.18 ± 0.99
DPPH IC50 (mg/mL)	14.03 ± 2.40	12.50 ± 1.30	2.72 ± 0.46 *	3.96 ± 0.39 *

Values are the mean (*n* = 6) ± standard deviation. * Mean values in the same line and the same species showed significant differences (*p* < 0.05).

**Table 3:** Phenolic compounds from leaves and stems of *J. cinerea* and *J. cordata* identified by ESI-IT-MS/MS.

	Compounds	Precursor Ion	Fragment Ions	*J. cinerea*		*J. cordata*	
		[M-H]-*m*/*z*	ESI-MS^N^ (*m*/*z*)	Leaves	Stems	Leaves	Stems
1	3,4-Dihydroxy-benzoic acid [33]	153	109, 153, 108	*	*	*	*
2	4-Hydroxy-benzoic acid °	137	92, 93, 136, 137	*	*	*	*
3	Caffeic acid °	179	135	*	*	*	*
4	Ellagic acid [34]	301	300.9, 229, 257	*	*	*	*
5	Gallic acid °	169	125	*	*	*	*
6	Gentisic acid °	153	109	*	*	*	*
7	*p*-coumaric acid °	163	119	*	*	*	*
8	Syringic acid °	197	182, 153	ND	*	ND	ND
9	Sinapic acid °	223	164, 179	ND	ND	ND	*
10	Tannic acid [35]	1700	1700	*	*	*	*
11	Vanillic acid °	167	157, 123, 108	ND	*	ND	*
12	Apigenin °	269	227, 150, 117	*	*	*	*
13	Catechol [36]	109	108, 91	*	*	*	*
14	Epicatechin °	289	245, 205, 179	ND	*	ND	*
15	Apigenin-7-*O*-Rutinoside [37]	577	269, 577	*	ND	ND	ND
16	Rutin hydrate [38]	609	301, 151	ND	ND	*	*
17	Isovitexin [39]	431	311, 341, 413	*	*	*	*
18	Pyrogalol [39]	125	124, 125	ND	ND	ND	*
19	Quercetin °	301	151, 179	ND	*	*	*
20	Rhoifolin [39]	577	577.1, 269, 268	*	ND	ND	ND
21	Rutin °	609	301, 179,151	ND	*	*	*
22	Vitexin [39]	431	311,341	*	ND	*	*

° Confirmed by authentic chemical standards. ND: Not detected. * Detected.

**Table 4:** Phenolic compounds content in methanolic extracts of leaves and stems from *J. cinerea* and *J. cordata* by HPLC-DAD (μg/g of sample).

Compounds	*J. cinerea*		*J. cordata*	
	Leaves	Stems	Leaves	Stems
4-Hydroxybenzoic acid	60.34 ± 0.50 *	35.97 ± 0.10 *	75.74 ± 0.89 *	22.09 ± 0.62 *
Caffeic acid	61.27 ± 0.10 *	22.19 ± 0.10 *	58.75 ± 0.31 *	50.49 ± 0.31 *
Gallic acid	261.68 ± 0.40 *	113.77 ± 0.60 *	284.45 ± 1.27 *	215.31 ± 1.19 *
Gentisic acid	634.55 ± 1.80 *	26.91 ± 0.40 *	864.62 ± 5.24 *	14.6 ± 0.21 *
*p*-coumaric acid	175.27 ± 0.40 *	62.98 ± 0.50 *	123.91 ± 0.13 *	ND
Sinapic acid	ND	ND	ND	12.44 ± 0.12 *
Syringic acid	ND	39.15 ± 0.60 *	ND	ND
Vanillic acid	ND	38.48 ± 0.10 *	ND	40.81 ± 0.80 *
Apigenin	148.06 ± 1.30 *	94.18 ± 1.80 *	343.54 ± 2.96 *	87.61 ± 1.46 *
Epicatechin	ND	230.05 ± 4.60 *	ND	90.16 ± 6.79 *
Quercetin	ND	198.52 ± 0.20 *	238.12 ± 1.63 *	573.98 ± 2.11 *

Values represent means (*n* = 9) ± standard deviation. * Mean values in the same line and the same species showed significant differences (*p* < 0.05). ND: No Detected.

## Data Availability

Not applicable.
